# Further evidence for epidemiological transition hypothesis for elderly suicides

**DOI:** 10.5249/jivr.v3i1.71

**Published:** 2011-01

**Authors:** Ajit Shah

**Affiliations:** ^a^International School for Communities, Rights and Inclusion, University of Central Lancashire, Preston, United Kingdom and West London Mental Health NHS Trust, London, United Kingdom.

## Abstract

**Background:**

A developmental model of epidemiological transition for elderly suicide rates with four sequential stages has been developed to simultaneously explain cross-national variations in elderly suicide rates, trends over time for elderly suicide rates and age-associated trends in suicides rates reported in the literature. This model was supported by demonstration of a curvilinear (inverted U-shaped curve) relationship between elderly suicide rates and socio-economic status fitting the quadratic equation Y = A + BX - CX2 (where Y is the suicide rate, X is the socio-economic status and A,B, and C are constants) in both sexes. However, this relationship was derived from a cross-sectional study and, therefore, only an association can be inferred. One way to substantiate this further would be to examine the above curvilinear relationship between suicide rates and socio-economic status in a series of younger age-bands because a large part of the epidemiological transition hypothesis was contingent upon the impact of socio-economic status, through a series of mechanisms, on life expectancy. It was hypothesized that the curvilinear (inverted U-shaped curve) relationship between suicide rates and socio-economic status would be absent in younger age-bands and may be present in the younger age-bands closer to the older age-bands (i.e. 45-54 years and 55-64 years).

**Methods:**

The curvilinear relationship between suicide rates in five age-bands 15-24 years to 55-64 years in both sexes and gross national domestic product (GDP), a measure of socio-economic status, fitting the above quadratic equation was examined with curve estimation regression model using data from the World Health Organization.

**Results:**

In males in the age-bands 35-44 years, 45-54 years and 55-64 years there was a statistically significant curvilinear (inverted U-shaped curve) relationship with GDP and fitted the quadratic equation Y = A + BX - CX2; this relationship was absent in males in the age-bands 15-24 years and 25-34 years. In females in the age-bands 45-54 years and 55-64 years there was a statistically significant curvilinear with GDP (inverted U-shaped curve) and fitted the quadratic equation Y = A + BX - CX2; this relationship was absent in females in the age-bands 15-24 years, 25-34 years and 35-44 years.

**Conclusions:**

Although caution should be exercised in accepting the model of the epidemiological transition hypothesis for elderly suicide rates because it had been generated from cross-sectional data using an ecological design, the findings of the current study of suicide rates in younger age-bands provide support for this hypothesis.

## Introduction

A developmental model of epidemiological transition for elderly suicide rates with four sequential stages has been developed to simultaneously explain cross-national variations in elderly suicide rates, trends over time for elderly suicide rates and age-associated trends in suicides rates reported in the literature.^1,2^This model suggests that each country would sequentially progress through the four stages over time with its socio-economic development, although different countries will be at different stages of development within this model. A curvilinear (inverted U-shaped curve) relationship between elderly suicide rates (defined as suicide rates for those aged 65-74 years and 75+ years) and socio-economic status fitting the quadratic equation Y = A + BX - CX2 (where Y is the suicide rate, X is the socio-economic status and A,B, and C are constants) has been demonstrated for both sexes to support this model.^2^The four stages of the model include: (i) low elderly suicide rate-low socio-economic society stage; (ii) high elderly suicide rate-low socio-economic society stage; (iii) high elderly suicide rate-high socio-economic society stage; and (iv) low elderly suicide rate-high socio-economic society stage. An explanatory model for each stage is provided below.

**Low elderly suicide rate-low socio-economic society stage**

Cross-national studies have observed that low socio-economic status is associated with low rates of suicide in the general population.^3,4^Cross-national and within country studies have also observed that higher income inequality is associated with lower suicide rates in the general population^6,7^and in the elderly.^5^Healthcare services are likely to be poorly developed in societies with low socio-economic status^5,8-12^and poorly developed healthcare services may lead to an increase in child mortality rates by being unable to provide primary preventative measures for diseases in childhood (e.g. immunization programs) and treatment for diseases that are directly related to low socio-economic status (e.g. infectious diseases).^13^Countries with low socio-economic status and higher income inequality have higher child mortality rates.^3,5,8^Increased child mortality rates is likely to lead to reduced life expectancy.^3,5,8^Suicide rates generally increase with age^14^and reduced life expectancy will result in fewer people reaching the age of increased risk for suicide in societies with low socio-economic status. This will result in a reduced number of elderly suicides in countries with low socio-economic status.^5,15^Additionally, selective survival of those at reduced risk of suicide in old age due to genetic or constitutional factors may further compound this trend^13^Also, in societies with low socio-economic status, those who do survive into old age may be at reduced risk of suicide because they may be able to better tolerate additional hardship in old age due to life-long exposure to adversity.^16-19^

**High elderly suicide rate-low socio-economic society stage**

Improvement in the socio-economic status of countries leads to the development of improved healthcare services.^3,5,8-12^This, in turn, may facilitate reduction in child mortality rates because of improved ability to provide primary preventative measures for diseases in childhood (e.g. immunization programs) and treatment for diseases that are directly related to low socio-economic status (e.g. infectious diseases).^13^This reduction in child mortality rates will lead to increased life expectancy.^3,5,8,15^This will result in greater number of individuals reaching the age of increased risk of suicide and an increase in elderly suicide rates because there is evidence of a positive correlation between elderly population size and elderly suicide rates.^5^This will lead to a gradual transition from low elderly suicide rate-low socio-economic society stage to a high elderly suicide rate-low socio-economic society stage.

**High elderly suicide rate-high socio-economic society stage**

Further socio-economic development will change societies from being socio-economically less developed to being socio-economically more developed.^3,5,8-12^This will lead to further improvement in healthcare services. This, in turn, will facilitate further reductions in child mortality rates because of improved ability to provide primary preventative measures for diseases in childhood (e.g. immunization programs) and treatment for diseases that are directly related to low socio-economic status (e.g. infectious diseases).^13^This further reduction in child mortality rates will further increase the life expectancy.^3,5,8,15^This will result in increasing number of individuals reaching the age of increased risk of suicide because there is evidence of a positive correlation between elderly population size and elderly suicide rates.^5^Both reduced child mortality rates and increased life expectancy will also lead to reduction in selective survival of those at reduced risk of suicide in old age due to constitutional or genetic factors. Also, the protective effects of life-long adversity on elderly suicide rates^16-19^is likely to be absent in countries with higher socio-economic status. Collectively, these changes will increase the number of individuals at increased risk of suicide in old age in socio-economically more developed societies. This will lead to a gradual transition from the high elderly suicide rate-low socio-economic society stage to a high elderly suicide rate-high socio-economic society stage.

**Low elderly suicide rate-high socio-economic society stage**

Theoretically, in socio-economically well developed societies, due to further reduction in child mortality rates and increase in life expectancy, greater number of people would reach the age of increased of suicide, and consequently lead to higher elderly suicide rates. However, in many socio-economically well developed countries elderly suicide rates are comparatively low^20^and they have declined over time.^5^Elderly suicide rates may progressively decline over many years in socio-economically very well developed societies due to improved efforts to control the risk factors for elderly suicides, enhance protective factors for elderly suicides, advances in medical care, prompt resuscitation of those who attempt suicide, better provision of healthcare (including mental health) services and public health initiatives to reduce suicide rates.^21-27^This will lead to a gradual transition from a high elderly suicide rate-high socio-economic society stage to a low elderly suicide rate-high socio-economic society stage.

The above epidemiological transition hypothesis is based upon socio-economic status of societies and the consequences of socio-economic status, but there may also be many other factors that interact with socio-economic status and also act independently to influence suicide rates. The above epidemiological model is supported by demonstration of a curvilinear (inverted U-shaped curve) relationship between elderly suicide rates (defined as suicide rates for those aged 65-74 years and 75+ years) and socio-economic status fitting the quadratic equation Y = A + BX - CX2 (where Y is the suicide rate, X is the socio-economic status and A,B, and C are constants) in both sexes.^2^However, this relationship was derived from a cross-sectional study and, therefore, only an association can be inferred.^2^The causal relationship and the direction of causality cannot be proved from a cross-sectional study.^2^One way to investigate this further would be to follow individual countries longitudinally over time as they progress through different socio-economic stages. However, such studies would be expensive and time consuming as they would require several decades. Another approach to substantiate the epidemiological transition hypothesis of elderly suicides would be to examine the above curvilinear relationship between suicide rates and socio-economic status in a series of younger age-bands (the five 10-year age-bands 15-24 years to 55-64 years) because a large part of the epidemiological transition hypothesis was contingent upon the impact of socio-economic status, through a series of mechanisms, on life expectancy. If the epidemiological transition hypothesis and its explanatory models are accurate then it is likely that this relationship will be absent in younger age-bands and would be more likely to be present in the older age-bands. It was hypothesized that the curvilinear (inverted U-shaped curve) relationship between suicide rates and socio-economic status would be absent in younger age-bands and may be present in the younger age-bands closer to the older age-bands (i.e. 45-54 years and 55-64 years). It is important to test this hypothesis because it may have implications for identification of potential risk and protective factors in the context of prevention programs.

## Methods

**Suicide rates**

Data on suicide rates for males and females in the five 10-year age-bands 15-24 to 54-65 years was ascertained from the WHO (http://www.who.int/whosis/database/mort/ table1.cfm). For a small number of countries only the raw figures for the number of suicides were available from the WHO website. Suicide rates for such countries were calculated by dividing the number of reported suicides by the population size in the relevant age-band and sex group available on the same website. Data were ascertained for the latest available year. The median (range) for the latest year of the suicide rate data was 2005 (1970-2007).

**Data on socio-economic status and other variables**

Data on the per capita Gross national domestic product (GDP), a proxy measure for socio-economic status of countries^3,5,21^was ascertained from the WHO website (http://www.who.int/countries/afg/en/) for the year 2006.

**Data analysis**

The relationship between suicide rates in each of five 10-year age-bands for both sexes and GDP was examined with curve estimation regression model in order to test the “a priori” hypothesis of a curvilinear relationship (inverted U-shaped curve) fitting the quadratic equation Y = A + BX - CX2 (where Y is the suicide rate, X is the GDP, and A,B, and C are constants).

## Results

Data on both suicide rates and the GDP were available for 102 countries from the WHO website.Table 1illustrates the characteristics of the curve estimation regression models for both sexes in all the five age-bands from 15-24 years to 55-64 years. In males in the age-bands 35-44 years, 45-54 years and 55-64 years there was a statistically significant curvilinear (inverted U-shaped curve) relationship with GDP and fitted the quadratic equation Y = A + BX - CX2; this relationship was absent in males in the age-bands 15-24 years and 25-34 years. In females in the age-bands  45-54 years and 55-64 years there was a statistically significant curvilinear with GDP (inverted U-shaped curve) and fitted the quadratic equation Y = A + BX - CX2; this relationship was absent in females in the age-bands 15-24 years, 25-34 years and 35-44 years.

**Table 1:Curve estimation regression models for the relationship between suicide rates and the GDP:** 

	R2	Degrees of Freedom	F Value	Significance	Regression Equation Y = A + BX – CX2
Males:					
15-24 years	0.036	102	1.89	NS	Y = 7.66+ 0X – 5.18e-9X2
25-34 years	0.029	102	1.51	NS	Y = 12.13+ 0.001X – 1.37e-8X2
35-44 years	0.07	102	3.81	P=0.025	Y = 9.97 + 0.001X – 2.07e-8X2
45-54 years	0.083	102	4.61	P=0.012	Y = 8.52 + 0.002X – 2.84e-8X2
55-64 years	0.11	102	6.51	P=0.002	Y = 7.96 + 0.002X – 3.45e-8X2
Females:					
15-24 years	0.006	102	0.31	NS	Y = 3.90 – 7.75e-6X - 2.84e-10X2
25-34 years	0.055	102	2.98	NS	Y = 2.35 + 0X – 1.38e-9X2
35-44 years	0.054	102	2.91	NS	Y = 3.35 + 9.16e-5X – 2.38e-10X2
45-54 years	0.22	102	14.49	P<0.0001	Y = 0.73 + 0X – 5.77e-9X2
55-64 years	0.138	102	8.15	P=0.001	Y = 1.55 + 0X – 5.79e-9X2

Y = Suicide rates / X = GDP / A, B and C are constants / NS=Not significant

## Discussion

Some methodological issues need consideration. Cross-national data on suicide rates should be viewed cautiously because: data were not available from all countries;^28,29^the validity of this data was unclear;^29,30^the legal criteria for the proof of suicide vary between countries and in different regions within a country;^29,31^some countries, particularly low-income countries, may have poor death registration facilities;^31^and, cultural and religious factors and stigma attached to suicide may lead to under-reporting of suicides.^29,32^Data on socio-economic status should also be viewed with caution because: the validity of this data is unclear; some countries may have poor infrastructure for providing accurate financial data;^5^and, their influence on social distribution of risk factors may be complex.^33^These latter concerns are more likely to be observed in low-income countries. However, data were gathered from the WHO data bank and were the latest and best available data set.

A large part of the epidemiological transition hypothesis described in detail in the Introduction was contingent upon the impact of socio-economic status, through a series of mechanisms, on life expectancy. Thus, it was hypothesized that the curvilinear (inverted U-shaped curve) relationship between suicide rates and socio-economic status would be absent in younger age-bands and may be present in the younger age-bands closer to the older age-bands (i.e. 45-54 years and 55-64 years). The presence of a curvilinear relationship between socio-economic status and age-bands 45-54 years and 55-64 years in both sexes and its absence in the age-bands 15-24 years and 25-34 years in both sexes is consistent with the previously developed epidemiological transition hypothesis of elderly suicides. Essentially as age-bands increased in age, the relationship between suicide rates and GDP became curvilinear (inverted U-shaped curve). Although caution should be exercised in accepting the model of the epidemiological transition hypothesis for elderly suicide rates because it had been generated from cross-sectional data using an ecological design, the findings of the current study of suicide rates in younger age-bands provide support for this hypothesis. The findings have important implications for identification of risk and protective factors for elderly suicides in the context of prevention programs as societies evolve through different soci0-economic stages.
